# Dry Eye Symptoms and Ocular Discomfort Among Palestinian University Students With and Without Contact Lens Use: A Cross‐Sectional Study

**DOI:** 10.1155/bmri/7797861

**Published:** 2026-10-17

**Authors:** Lemar Edhilie, Ayah Yousef, Faten Dawoud, Bisan Ziyada, Hamza Abualhasan, Mustafa Ghanim, Maha Rabayaa, Malik Alqub, Mohammad Abuawad, Ithar M. Beshtawi

**Affiliations:** ^1^ Department of Medicine Faculty of Medicine and Allied Medical Sciences An-Najah National University Nablus State of Palestine najah.edu; ^2^ Department of Biomedical Sciences and Basic Clinical Skills Faculty of Medicine and Allied Medical Sciences An-Najah National University Nablus State of Palestine najah.edu; ^3^ Optometry Department Faculty of Medicine and Allied Medical Sciences An-Najah National University Nablus State of Palestine najah.edu

**Keywords:** CLDEQ-8, contact lenses, DES, dry eye symptoms, ocular discomfort, OSDI, university students

## Abstract

**Background:**

Ocular dryness and discomfort are commonly reported by contact lens wearers, particularly among young adults. This study evaluated self‐reported ocular symptoms among Palestinian university students and compared contact lens users with nonusers.

**Methods:**

A cross‐sectional online survey was conducted among Palestinian university students using dry eye symptom (DES) items, the Contact Lens Dry Eye Questionnaire‐8 (CLDEQ‐8), and the Ocular Surface Disease Index (OSDI). Descriptive, comparative, and multivariable analyses were performed to examine ocular symptoms and their associations with contact lens use and relevant participant characteristics.

**Results:**

A total of 408 students participated, of whom 202 (49.5%) were contact lens users and 87.7% were female. Dryness and sandy/gritty sensation were reported significantly more frequently by contact lens users than nonusers (both *p* < 0.001). Among contact lens users, 82.7% had OSDI scores ≥ 13, compared with 78.1% of nonusers; however, neither OSDI symptom‐category distribution (*p* = 0.496) nor mean OSDI score (29.18 ± 19.05 vs. 26.84 ± 17.65; *p* = 0.20) differed significantly between groups. Among contact lens users, CLDEQ‐8 responses indicated a high burden of lens‐related discomfort and dryness. In multivariable analysis, contact lens use was not independently associated with OSDI score after adjustment for gender and daily screen exposure (B = 2.15, 95% CI −1.46 to 5.77; *p* = 0.242). Participants reporting > 8 h/day of screen exposure had significantly higher OSDI scores than those reporting 2–4 h/day (B = 10.37, 95% CI 0.45 to 20.29; *p* = 0.040).

**Conclusions:**

Self‐reported ocular dryness and discomfort were common among Palestinian university students. Contact lens users reported some ocular symptoms more frequently and experienced substantial lens‐related discomfort, but contact lens use was not independently associated with greater overall OSDI symptom burden after adjustment for gender and screen exposure. Because objective tear‐film and ocular‐surface assessments were not performed, these findings should not be interpreted as establishing dry eye disease or attributing the reported symptoms specifically to contact lens wear.

## 1. Introduction

Dry eye disease (DED) is a prevalent multifactorial condition of the ocular surface, characterized by tear film instability, raised osmolarity, ocular surface inflammation, and neurosensory dysfunction. DED is linked to ocular discomfort, visual impairments, and possible damage to the ocular surface [1]. The global prevalence of DED exhibits significant variability, ranging from around 5% to 50%, influenced by geographic location, age, and sex [2–4]. DED is linked with a significant public health and financial burden, with sources predicting that the United States spends $3.8 billion per year on treatment. Physician visits and productivity loss add to the total societal cost, which was estimated at $55.4 billion per year in the US over a decade ago [5]. Significant risk factors for DED included advancing age, contact lens wear, increased digital device screen exposure, and higher psychological stress [6]. Furthermore, the global burden of DED is anticipated to increase, which constitutes a significant public health problem with the world′s aging population and the increasingly prevalent use of digital screens among pediatric and young adult populations [7]. Among young adults, particularly university students, dry eye symptoms (DES) may be influenced by multiple behavioral and environmental factors, including contact lens wear, prolonged digital screen exposure, and sustained near‐visual tasks. These exposures may contribute to altered blinking, increased tear evaporation, tear‐film instability, and ocular surface dryness [8–10]. Given its increasing prevalence and impact within this population, DED is increasingly recognized as a public health concern, as its symptoms can affect quality of life and reduce productivity at both individual and societal levels [8–10].

Contact lenses are widely used for refractive correction worldwide, with more than 140 million wearers globally, compared with approximately 70 million in the late 1990s [11]. Among young adult contact lens users, ocular dryness and discomfort are frequently reported and may contribute to reduced wearing time or contact lens discontinuation [12, 13]. According to estimates, 15% to 55% of contact lens wearers suffer symptoms consistent with dry eye, which is higher than the rate reported in noncontact lens wearers [14–18]. Questionnaire‐based studies have reported a considerable burden of ocular dryness and discomfort among contact lens wearers. In Saudi Arabia, approximately one‐third of contact lens users reported DES, with symptom burden associated with factors such as lens‐wearing duration, environmental conditions, and lens‐care practices [2, 19]. Other factors, including prolonged screen exposure, smoking, and female sex, have also been associated with greater DES burden [2, 20]. Studies among university students in Thailand [21] and Brazil [22] have similarly reported associations between contact lens wear, prolonged screen exposure, and DES or questionnaire‐based dry eye classifications. Malaysian university students who wore contact lenses also reported more frequent and severe ocular symptoms, particularly with prolonged computer use [23], whereas recent studies from Syria and Jordan have documented a high frequency of self‐reported ocular dryness and related symptoms among contact lens wearers [24, 25].

Importantly, the presence of dryness or ocular discomfort in a contact lens wearer does not by itself establish DED or demonstrate that the symptoms were caused by contact lens wear. Such symptoms may arise from contact lens‐related discomfort, underlying DED, or other ocular or visual conditions. Accordingly, symptom questionnaires are useful for characterizing symptom burden but, in the absence of appropriate clinical assessment and differential diagnosis, cannot determine the underlying cause of these symptoms [12].

Research on ocular dryness and discomfort among contact lens users has increased globally and regionally; however, evidence from Palestine remains limited. Previous Palestinian studies have examined questionnaire‐reported DESs in the general population and their association with factors such as digital device use among university students [26, 27]. A study among nurses in the northern West Bank also reported a high prevalence of symptomatic dry eye and an association between contact lens use and symptom reporting [28]. More recently, increased screen exposure, inadequate sleep, and contact lens use were associated with greater DES burden among Palestinian university students [27]. However, these studies did not specifically examine contact lens‐related symptom patterns together with lens‐wearing duration, lens type, and lens‐care practices among Palestinian university students.

Thus, this study is aimed at evaluating the prevalence and severity of self‐reported DES and ocular discomfort among Palestinian university students, and comparing these symptoms between contact lens users and nonusers. The study also examined contact lens use patterns and practices and explored demographic and behavioral factors associated with DES burden.

## 2. Methodology

### 2.1. Study Design and Sampling

This study used a descriptive cross‐sectional design to evaluate the prevalence and severity of self‐reported DES and associated factors among Palestinian university students. The research was carried out from July to October 2025. The study targeted Palestinian university students residing in Palestine and enrolled in accredited Palestinian universities (An‐Najah National University, Palestine Technical University–Kadoorie, Birzeit University, Arab American University, and Al‐Quds University). Eligible participants were required to meet the following inclusion criteria: Palestinian students currently enrolled in an accredited university within Palestine. Those who did not meet the inclusion criteria were excluded, and individuals with ocular or systemic diseases, a history of eye surgery, or who used medications known to cause dry eye were excluded from the study.

The questionnaire was designed using Google Forms and distributed electronically via student sites on social media, student emails, and universities′ e‐teaching tools. A convenient sampling method was used, taking into consideration that the total number of Palestinian university students is approximately 139,000 students. The sample size was calculated at a 95% confidence interval (CI) and accepted a default margin of error of 5%. The minimal sample size was calculated as 383 participants using an online sample size calculator (http://www.raosoft.com). Then, a total of 408 participants who met the inclusion criteria completed the survey and enrolled in the study.

### 2.2. Data Collection Tools

The study used a self‐administered questionnaire adapted from a previously published study, which was modified and adjusted to suit the objectives of our current research [2]. To ensure the linguistic and conceptual accuracy of the questionnaire, a back‐translation process was conducted: the questionnaire was first translated from English into Arabic by a translator, then retranslated into English by another independent translator to ensure that the original meaning was preserved.

The final questionnaire consisted of four sections: the first covered sociodemographic information such as age, history of ocular diseases, and contact lens use and frequency; the second assessed DES; the third included the Contact Lens Dry Eye Questionnaire‐8 (CLDEQ‐8) [29]; and the fourth comprised the Ocular Surface Disease Index (OSDI) [30].

Furthermore, a pilot study on a sample of 20 participants was administered to evaluate the clarity of the questionnaire elements, validity, and the time required to complete the questionnaire. According to the results in, it was found that higher values of the Cronbach′s coefficient (*α*) reflect a better level of internal consistency, with values of ≥ 0.80 being an indicator of good to excellent reliability, also found that all corrected correlation coefficients between the items and the total were ≥ 0.30, which are considered acceptable values and indicate that each item clearly contributes to the structure measured by the scale.

To minimize sampling bias, we distributed the questionnaire to several different universities, and during distribution, we clearly stated the basic criteria required for participation without mentioning disease‐related symptoms. To reduce the response bias, the confidentiality of the participants′ identities was ensured to encourage accurate and truthful answers. Validated tools such as OSDI and CLDEQ‐8 were carefully translated and back‐translated, and a pilot study was conducted to reduce information bias to a minimum. Finally, potential confounding factors were addressed by analyzing associations between key behavioral and demographic variables.

### 2.3. Ethical Approval

Ethical approval to conduct this study was granted by the Institutional Review Board (IRB) at An‐Najah National University, Nablus, Palestine (IRB Ref: Med. Dec.2024/33). The study adhered to the ethical principles outlined in the Declaration of Helsinki for research involving human participants. Electronic informed consent was obtained from all participants prior to enrollment. The consent form clearly described the purpose of the study and assured participants of the anonymity of their responses and the confidentiality of all collected data.

### 2.4. Statistical Analysis

Statistical analyses were conducted utilizing the Statistical Package for the Social Sciences Statistics (SPSS) for Windows, Version 21 (IBM Corp., Armonk, New York, United States). The OSDI was used to assess self‐reported ocular symptoms and related vision‐specific functional impairment and was not considered a diagnostic test for DED. OSDI scores were categorized as 0–12 (normal range), 13–22 (mild symptoms), 23–32 (moderate symptoms), and > 32 (severe symptoms). For analyses restricted to contact lens users, participants with an OSDI score ≥ 13 were classified as having OSDI‐defined ocular symptoms, whereas those with an OSDI score < 13 were classified within the normal OSDI range. Because OSDI responses may overlap with symptoms arising from other ocular or visual conditions, and objective tear‐film or ocular‐surface assessments were not performed, these categories were interpreted as symptom‐based classifications rather than as a diagnosis of dry eye [30]. Descriptive analysis based on the frequency and percentage was done for all variables, including contact lens users′ demographic data, and lens use frequency and practices. Also, the frequency distribution for DES questions and CLDEQ‐8 was tabulated. Cross‐tabulation was used to assess the association between the demographic data and the use of contact lenses, and the association between OSDI‐defined ocular symptom status among contact lens users and their demographic characteristics and contact lens‐related practices. Relations were tested using the chi‐square test. The association between contact lens use and OSDI score was further evaluated using multivariable linear regression, with OSDI score as the dependent variable and gender, contact lens use, and daily screen exposure (reference category: 2–4 h/day) as independent variables. Multicollinearity was assessed using tolerance and the variance inflation factor (VIF). Regression coefficients (B), 95% CIs, and *p* − values were reported. Statistical significance was set at *p* < 0.05.

## 3. Results

### 3.1. Participants Basic Characteristics

A total of 408 students had been enrolled in the study. Among the total sample, 87.7% of the participants were females. 52.5% were from villages and 45.5% were from cities. More than half of the participants were studying programs in the medical fields, 26.7% were studying scientific nonmedical programs, and 16.9% were studying humanities and art‐related programs. 42.7% of the participants reported exposure to screens for more than 8 h per day, and 34.3% were exposed between 6 and 8 h. Regarding the use of contact lenses, 49.5% of the participants reported using contact lenses. Results are shown in Table 1.

**Table 1:** Description of the total sample basic characteristics (*n* = 408).

Variable	Frequency (%)
Gender	
Male	50 (12.3)
Female	358 (87.7)
Place of residence	
Village	214 (52.5)
City	186 (45.5)
Camp	8 (2)
Field of study	
Medical	230 (56.4)
Scientific nonmedical	109 (26.7)
Humanities and art	69 (16.9)
Daily exposure to screens	
2–4 h	14 (3.4)
4–6 h	80 (19.6)
6–8 h	140 (34.3)
> 8 h	174 (42.7)
Do you use contact lenses?	
Yes	202 (49.5)
No	206 (50.5)

### 3.2. The Association Between Participants′ Basic Characteristics and the Use of Contact Lenses

Table 2 shows the association between participants′ characteristics and the use of contact lenses. Gender was significantly associated with contact lens use (*p* − value < 0.001), with a higher proportion of females (53.1%) using contact lenses compared to males (24%). The other variables, including place of residence, field of study, and daily screen exposure time, were not significantly associated with contact lens usage (*p* − value > 0.05).

**Table 2:** The association between participants′ basic characteristics and the use of contact lenses.

Variable	Do you use contact lenses?		*p*
	Yes, *n* (%)	No, *n* (%)	
Gender			
Male	12 (24)	38 (76)	**< 0.001**
Female	190 (53.1)	168 (46.9)	
Place of residence			
Village	100 (46.7)	114 (53.3)	0.423
City	97 (52.2)	89 (47.8)	
Camp	5 (62.5)	3 (37.5)	
Field of study			
Medical	108 (47)	122 (53)	0.276
Scientific nonmedical	54 (49.5)	55 (50.5)	
Humanities	40 (58)	29 (42)	
Daily exposure to screens			
2–4 h	7 (50)	7 (50)	0.273
4–6 h	32 (40)	48 (60)	
6–8 h	75 (53.6)	65 (46.4)	
> 8 h	88 (50.6)	86 (49.4)	

*Note:* The statistically significant value is shown in bold.

### 3.3. Description of the Lens Type, Frequency of Use, Dependence and Lens‐Related Hygiene Practices Among Contact Lens Users

The majority of contact lens users reported using soft lenses (96.5%). More than half of the contact lens users were using them 4–8 h daily. Regarding the extent of the dependence on contact lenses, 42.1% of the contact lens users reported using them most of the time, 26.7% were using them sometimes, 15.8% were using them all the time, 15.4% were using contact lenses rarely. 70.8% of the contact lens users reported proper hygiene practices, presented as hand washing and changing the lens solution after each use, as shown in Table 3.

**Table 3:** Description of the lens type, frequency of use, dependence and lens‐related hygiene practices among contact lens users (*n* = 202).

Variable	Frequency (%)
The type of lens	
Soft	195 (96.5)
Rigid	5 (2.5)
Others	2 (1)
Frequency of use (h/day)	
< 4 h	21 (10.4)
4–8 h	124 (61.4)
8–12 h	48 (23.8)
> 12 h	9 (4.4)
To what extent do you depend on contact lenses in your daily activities?	
Rarely	31 (15.4)
Sometimes	54 (26.7)
Most of the time	85 (42.1)
All the time	32 (15.8)
Do you wash your hands and change the lens solution after each use?	
No	16 (7.9)
Yes	143 (70.8)
Sometimes	43 (21.3)

### 3.4. The Association Between DES and Using Contact Lenses

The association between using contact lenses and DES is shown in Table 4. The dryness in the eyes and the feeling of a sandy or gritty sensation were significantly associated with using contact lenses (*p* − value < 0.001). Among the contact lens users, 26.7% reported often dryness of the eyes compared with 18% among nonusers. Although 18.4% of contact lens nonusers had not reported dryness in the eyes, only 5% of the contact lens users reported no dryness in the eyes. A total of 22.3% of the contact lens users reported often having a sandy or gritty sensation in their eyes, compared with only 9.7% of nonusers. A total of 39.8% of the nonusers had never had sandy or gritty sensations in their eyes, whereas only 23.3% of the contact lens users had never experienced the sandy or gritty sensation. The other DES were not significantly different between contact lens users and nonusers (*p* − value > 0.05). The remaining symptoms were reported as follows: a burning sensation affected 89.6% of contact lens users and 81.6% of nonusers. Eye redness occurred in 79.2% of contact lens users versus 75.2% of nonusers. Debris on the eyelashes was noted in 64.4% of contact lens users and 55.8% of nonusers, whereas morning eyelid stickiness was reported by 38.6% versus 35%, respectively. However, none of these differences between contact lens users and nonusers were statistically significant (*p* > 0.05).

**Table 4:** The association between dry eye symptoms (DES) and using contact lenses.

DES	Noncontact lens users, *n* (%)	Contact lens users, *n* (%)	*p*
DES‐Q1 (dryness)			
Never	38 (18.4)	10 (5)	**< 0.001**
Rarely	40 (19.4)	29 (14.4)	
Sometimes	66 (32)	78 (38.6)	
Often	37 (18)	54 (26.7)	
Always	25 (12.1)	31 (15.31)	
DES‐Q2 (sandy/gritty sensation)			
Never	82 (39.8)	47 (23.3)	**< 0.001**
Rarely	36 (17.5)	45 (22.3)	
Sometimes	61 (29.6)	59 (29.2)	
Often	20 (9.7)	45 (22.3)	
Always	7 (3.4)	6 (3)	
DES‐Q3 (burning sensation)			
Never	38 (18.4)	21 (10.4)	0.18
Rarely	43 (20.9)	51 (25.2)	
Sometimes	59 (28.6)	65 (32.2)	
Often	51 (24.8)	47 (23.3)	
Always	15 (7.3)	18 (8.9)	
DES‐Q4 (eye redness)			
Never	51 (24.8)	42 (20.8)	0.43
Rarely	54 (26.2)	45 (22.3)	
Sometimes	62 (30.1)	62 (30.7)	
Often	30 (14.6)	42 (20.8)	
Always	9 (4.4)	11 (5.4)	
DES‐Q5 (debris on eyelashes)			
Never	91 (44.2)	72 (35.6)	0.084
Rarely	49 (23.8)	58 (28.7)	
Sometimes	43 (20.9)	36 (17.8)	
Often	18 (8.7)	22 (10.9)	
Always	5 (2.4)	14 (6.9)	
DES‐Q6 (eyelid stickiness (morning))			
Never	134 (65)	124 (61.4)	0.601
Rarely	42 (20.4)	42 (20.8)	
Sometimes	20 (9.7)	18 (8.9)	
Often	9 (4.4)	16 (7.9)	
Always	1 (0.5)	2 (1)	

*Note:* Values with statistical significance are shown in bold.

### 3.5. Description of the Contact Lens‐Related Effects Among Contact Lens Users

Table 5 shows that 95% of the contact lens users experienced different degrees of eye discomfort while wearing contact lenses. For end‐of‐day discomfort intensity, 49.5%, 32.7%, and 3.5% selected response scores of 2, 3, and 4, respectively. Dryness was reported by 93.1% of contact lens users, while 36.1% and 5.4% selected response scores of 3 and 4, respectively, for end‐of‐day dryness intensity. Changes between clear and blurry or foggy vision were reported by 87.1% of contact lens users, while 26.2% and 2.0% selected response scores of 3 and 4, respectively, for how noticeable these changes were at the end of lens wear. Additionally, 88.1% of the participants reported that their eyes bothered them to the extent that they wanted to close them, and all participants reported needing to remove their contact lenses due to discomfort.

**Table 5:** Contact lens‐related effects among contact lens users (*n* = 202).

CLDEQ‐8	Frequency	%
1. During a typical day in the past 2 weeks, how often did your eyes feel discomfort while wearing your contact lenses?		
Never	10	5
Rarely	47	23.3
Sometimes	78	38.6
Frequently	56	27.7
Constantly	11	5.4
2. When your eyes felt discomfort with your contact lenses, how intense was this feeling of discomfort at the end of your wearing time?		
Not at all intense	29	14.4
2	100	49.5
3	66	32.7
4	7	3.5
Very intense	0	0
3. During a typical day in the past 2 weeks, how often did your eyes feel dry?		
Never	14	6.9
Rarely	42	20.8
Sometimes	78	38.6
Frequently	53	26.2
Constantly	15	7.4
4. When your eyes felt dry, how intense was this feeling of dryness at the end of your wearing time?		
Not at all intense	28	13.9
2	90	44.6
3	73	36.1
4	11	5.4
Very intense	0	0
5. During a typical day in the past 2 weeks, how often did your vision change between clear and blurry or foggy while wearing your contact lenses?		
Never	26	12.9
Rarely	62	30.7
Sometimes	70	34.7
Frequently	33	16.3
Constantly	11	5.4
6. When your vision was blurry, how noticeable was the changed blurry or foggy vision at the end of your wearing time?		
Not at all intense	43	21.3
2	102	50.5
3	53	26.2
4	4	2
Very intense	0	0
7. During a typical day in the past 2 weeks, how often did your eyes bother you so much that you wanted to close them?		
Never	24	11.9
Rarely	45	22.3
Sometimes	67	33.2
Frequently	46	22.8
Constantly	20	9.9
8. How often during the past 2 weeks did your eyes bother you so much while wearing the contact lenses that you felt as if you needed to stop whatever you were doing and take out your contact lenses?		
Never	0	0
Less than once a week	0	0
Weekly	106	52.5
Several times a week	67	33.2
Daily	17	8.4
Several times a day	12	5.9

### 3.6. DES Severity Based on the OSDI Scale

Table 6 shows that, among contact lens users, 17.3% had no/minimal symptoms according to the OSDI, whereas 31.2%, 15.3%, and 36.1% were classified as having mild, moderate, and severe symptom levels, respectively. Among nonusers, 21.9% had no/minimal symptoms according to the OSDI, whereas 25.2%, 16.0%, and 36.9% were classified as having mild, moderate, and severe symptom levels, respectively. Neither the distribution of OSDI symptom severity categories nor the mean OSDI score differed significantly between contact lens users and nonusers (*p* > 0.05).

**Table 6:** OSDI symptom severity categories among contact lens users and nonusers.

OSDI category	Contact lens users		Contact lens nonusers		*p*
	*n*	%	*N*	%	
No/minimal symptoms	35	17.3	45	21.9	0.496
Mild	63	31.2	52	25.2	
Moderate	31	15.3	33	16	
Severe	73	36.1	76	36.9	
Total	202	100	206	100	
OSDI score (out of 100)					
Mean ± SD	29.18 ± 19.05		26.84 ± 17.65		0.2

### 3.7. OSDI Among Contact Lens Users

During the preceding week, 79.2% of contact lens users reported sensitivity to light, 57.4% reported gritty eyes, 45.5% reported painful or sore eyes, 64.4% reported blurred vision, and 56.9% reported poor vision. Regarding limitations in daily activities due to eye problems, 65.8% had discomfort while reading, 36.1% had discomfort while driving at night, 82.7% had difficulties working with computers or digital devices, and 65.8% experienced problems while watching TV. Concerning situations associated with uncomfortable feelings, 87.1% experienced discomfort under windy conditions, 81.2% reported discomfort in very dry places, and 68.3% felt discomfort in air‐conditioned areas. Results are shown in Table 7.

**Table 7:** Ocular Surface Disease Index among contact lens users (*n* = 202).

OSDI	None of the time	Some of the time	Half of the time	Most of the time	All of the time
Eyes that are sensitive to light	42 (20.8)	82 (40.6)	34 (16.8)	30 (14.9)	14 (6.9)
Eyes that feel gritty	86 (42.6)	76 (37.6)	22 (10.9)	14 (6.9)	4 (2)
Painful or sore eyes	110 (54.5)	62 (30.7)	17 (8.4)	12 (5.9)	1 (0.5)
Blurred vision	72 (35.6)	87 (43.1)	21 (10.4)	16 (7.9)	6 (3)
Poor vision	87 (43.1)	71 (35.1)	24 (11.9)	14 (6.9)	6 (3)
Discomfort while reading	69 (34.2)	78 (38.6)	31 (15.3)	16 (7.9)	8 (4)
Discomfort while driving at night	129 (63.9)	37 (18.3)	20 (9.9)	11 (5.4)	5 (2.5)
Discomfort while working with a computer or digital devices	35 (17.3)	94 (46.5)	42 (20.8)	20 (9.9)	11 (5.4)
Discomfort while watching TV	69 (34.2)	79 (39.1)	30 (14.9)	16 (7.9)	8 (4)
Discomfort during windy conditions	26 (12.9)	68 (33.7)	32 (15.8)	48 (23.8)	28 (13.9)
Discomfort in places or areas with low humidity (very dry)	38 (18.8)	85 (42.1)	31 (15.3)	37 (18.3)	11 (5.4)
Discomfort in air‐conditioned areas	64 (31.7)	55 (27.2)	31 (15.3)	33 (16.3)	19 (9.4)

### 3.8. Factors Associated With OSDI‐Defined Ocular Symptoms Among Contact Lens Users

Based on the prespecified OSDI threshold, contact lens users were classified as having OSDI‐defined ocular symptoms when their OSDI score was ≥ 13 and as being within the normal OSDI range when their score was < 13. Associations between this classification and participants′ biodemographic characteristics and contact lens‐related practices were then evaluated. None of the studied variables was significantly associated with OSDI‐defined ocular symptoms (*p* > 0.05), as shown in Table 8.

**Table 8:** Factors associated with OSDI‐defined ocular symptoms among contact lens users.

Variable	OSDI‐defined ocular symptoms		*p*
	Normal OSDI range (< 13)	Elevated OSDI score (≥ 13)	
Gender			
Male	1 (8.3)	11 (91.7)	0.396
Female	34 (17.9)	156 (82.1)	
Place of residence			
Village	13 (13)	87 (87)	0.273
City	21 (21.6)	76 (78.4)	
Camp	1 (20)	4 (80)	
Field of study			
Medical	17 (15.7)	91 (84.30	0.801
Scientific nonmedical	10 (18.50	44 (81.5)	
Humanities and art	8 (20)	32 (80)	
Type of contact lens			
Soft	34 (17.4)	161 (82.6)	0.281
Rigid	0 (0)	5 (100)	
Others	1 (50)	1 (50)	
How many hours do you wear lenses per day?			
< 4 h	5 (23.8)	16 (76.2)	0.187
4–8 h	25 (20.2)	99 (79.8)	
8–12 h	5 (10.4)	43 (89.6)	
> 12 h	0 (0)	9 (100)	
To what extent do you depend on contact lenses in your daily activities?			
Rarely	9 (29)	22 (71)	0.055
Sometimes	9 (16.7)	45 (83.3)	
Most of the time	16 (18.8)	69 (81.2)	
All day	1 (3.1)	31 (96.9)	
Do you wash your hands before using contact lenses, and do you make sure to change the lens solution after each use?			
No	2 (12.5)	14 (87.5)	0.712
Yes	24 (16.8)	119 (83.2)	
Sometimes	9 (20.9)	34 (79.1)	

### 3.9. Multivariable Analysis of DES (OSDI Score)

In the multivariable linear regression model with total OSDI score as the dependent variable, contact lens use was not independently associated with DES severity after adjustment for gender and daily screen exposure (B = 2.15, 95% CI −1.46 to 5.77; *p* = 0.242). Gender was also not significantly associated with OSDI score (B = −0.89, 95% CI −6.38 to 4.60; *p* = 0.751). Compared with participants reporting 2–4 h of daily screen exposure, those reporting > 8 h/day had significantly higher OSDI scores (B = 10.37, 95% CI 0.45 to 20.29; *p* = 0.040), as shown in Table 9.

**Table 9:** Multivariable linear regression of OSDI score by gender, contact lens use, and screen time.

Predictor	B	Std. Error	Beta	T	*p*	95% CI (Lower–Upper)	Tolerance	VIF
Gender (female vs. male)	−0.886	2.793	−0.016	−0.317	0.751	−6.377 to 4.604	0.963	1.038
Contact lens use (yes vs. no)	2.154	1.84	0.059	1.171	0.242	−1.463 to 5.771	0.955	1.047
Screen time (4–6 h vs. 2–4 h)	2.787	5.263	0.06	0.53	0.597	−7.560 to 13.134	0.185	5.405
Screen time (6–8 h vs. 2–4 h)	5.612	5.09	0.145	1.102	0.271	−4.395 to 15.618	0.138	7.227
Screen time (> 8 h vs. 2–4 h)	10.371	5.045	0.279	2.056	**0.04**	0.454–20.288	0.13	7.703

*Note:* The statistically significant value is shown in bold.

## 4. Discussion

CLs can disrupt tear film stability, increase tear evaporation, and induce mechanical irritation of the ocular surface, leading to ocular dryness and discomfort and may ultimately lead to CL dropout [18, 31, 32]. Based on these mechanisms, this study evaluated self‐reported ocular dryness and discomfort among Palestinian university students and examined their relationship with contact lens use. The findings of this study showed that the majority of CL wearers (96.5%) use soft contact lenses. This finding is consistent with previously reported data in Palestine [33]. Additionally, the study showed that female participants were significantly more likely to wear CL than male participants (53.1% vs. 24.0%; *p* < 0.001). This pattern is consistent with findings reported in previous local [33], regional, and international studies, where contact lens use among young women is often influenced by practical needs and esthetic preferences [19, 21, 22, 26, 27, 34] In contrast, no statistically significant association was found between CL use and other demographic characteristics such as residency, field of study, or daily screen exposure.

The DES comparisons showed nonsignificant differences between contact lens users and nonusers for specific symptoms. However, symptoms of dryness (95%) and gritty sensation (76.7%) were statistically significantly higher in the CL users group (*p* < 0.001). Additionally, the results of the CLDEQ‐8 questionnaire showed that a high proportion of CL users experienced lens‐related discomfort (95.0%), with 41.5% selecting scores of 3 or 4 for end‐of‐day dryness intensity. Moreover, 88.1% of participants reported that their eyes became sufficiently uncomfortable that they needed to close them. Changes between clear and blurry or foggy vision during contact lens wear were reported by 87.1% of CL users. These findings are consistent with previous research demonstrating a strong association between CL wear and DES [35, 36]. Taken together, these findings suggest that CL users reported more frequent dryness and gritty sensation than nonusers, even though overall OSDI‐based symptom prevalence did not differ significantly between groups. However, because symptoms of dryness may arise from contact lens wear, from DED, or from other unrelated causes, these questionnaire findings describe the occurrence of symptoms only and do not establish their cause. Therefore, considering the high frequency of reported DES among CL users in this study and in previous research **[** **35**, **36** **]**, it is important to highlight the clinical relevance of lens‐related discomfort among young adults since these symptoms are considered among the main reasons for reducing daily wearing time and CL discontinuation.

When DES severity was assessed using the OSDI, the proportion of contact lens users with OSDI scores ≥ 13 (82.7%) and the mean OSDI score (29.2 ± 19.05) were higher than those observed among non‐contact lens users (78.1% and 26.84 ± 17.65, respectively), although these differences were not statistically significant. One possible explanation for the lack of statistical significance is that both groups were exposed to similar environmental and behavioral risk factors, particularly prolonged digital screen exposure, which is common among university students.

Importantly, the multivariable analysis showed that contact lens use was not independently associated with OSDI score after adjustment for gender and daily screen exposure. In contrast, participants reporting more than 8 h of daily screen exposure had significantly higher OSDI scores than those reporting 2–4 h per day. These findings indicate that the high burden of DESs observed among contact lens users should not be attributed solely to contact lens wear, as concurrent behavioral factors, particularly prolonged screen exposure, may also contribute to symptom severity.

The symptom burden observed among contact lens users in the present study was relatively high compared with that reported in several previous questionnaire‐based studies [2]. Such differences should be interpreted cautiously because studies vary in population characteristics, contact lens types and wearing patterns, symptom instruments, score thresholds, screen exposure, and environmental conditions. Importantly, questionnaire‐derived symptom estimates should not be interpreted as directly comparable estimates of clinically diagnosed DED.

Similarly, the proportion of participants with elevated OSDI scores and the mean OSDI score in the present study were higher than those reported in some previous studies from Palestine and neighboring countries [37]. Differences in study populations and behavioral exposures may partly explain this variation. The present sample consisted specifically of university students, among whom prolonged digital screen exposure was common. Extended screen use has been associated with altered blinking and ocular dryness [8]. No statistically significant association was observed between contact lens wearing duration or lens‐care practices and OSDI‐defined ocular symptom status in the present study.

Although OSDI scores did not differ significantly between contact lens users and nonusers, DES responses showed that dryness and sandy/gritty sensation were reported more frequently by contact lens users. Within the contact lens user group, CLDEQ‐8 responses also demonstrated a substantial burden of lens‐related discomfort and dryness during contact lens wear. This pattern has also been reported in previous studies where CL wear was found to play a significant role in ocular discomfort and dryness symptoms, even when no significant difference in the clinical signs of DED is observed between wearers and nonwearers [35]. These symptoms may negatively affect quality of life and may lead to discontinuation of CL use. The DES items assessed general ocular symptoms in both contact lens users and nonusers and were therefore not specific to contact lens wear. In contrast, the CLDEQ‐8 was specifically developed to assess symptoms experienced during soft contact lens wear, including dryness, discomfort, visual disturbance, and the need to remove lenses because of discomfort [29]. The OSDI assesses broader ocular symptoms, vision‐related functional impairment, and environmental triggers and is not specific to contact lens wear [30].

The present study has several strengths. It investigated the DED symptoms among CL users using validated instruments such as OSDI and CLDEQ‐8. Additionally, it included students from multiple universities in Palestine, which enhances the generalizability of the findings. However, several limitations should be noted. First, the cross‐sectional design of the study prevents the identification of causal relationships; it only identifies correlations. Second, the use of a convenience sampling method may result in sampling bias, and reliance on self‐reported data could introduce recall bias. The marked predominance of female participants may also limit the generalizability of the findings, particularly to male university students. Furthermore, the study′s results may have been influenced by the duration of screen use in both groups, indicating a potential confounding factor. Although the multivariable analysis adjusted for gender and daily screen exposure, residual confounding from other unmeasured behavioral, environmental, or contact lens‐related factors cannot be excluded. Finally, the absence of objective clinical assessments, such as tear film breakup time or the Schirmer test, limits the ability to correlate subjective symptoms with the clinical signs of dry eye. Consequently, this study cannot estimate the prevalence of clinically diagnosed DED or determine whether the reported symptoms were caused by contact lens wear, underlying DED, or other ocular or visual conditions.

Future long‐term studies should be conducted to evaluate the causal relationship between contact lens wear and the progression of DED. For a more comprehensive assessment, objective clinical tests such as tear film break‐up time and Schirmer′s test, along with symptom‐based questionnaires, should be incorporated. In addition, future research should investigate the combined effects of contact lens use and digital screen exposure within the target population, as these factors may interact and contribute to ocular surface symptoms in young adults.

## 5. Conclusions

DES were common among Palestinian university students, and contact lens users reported greater lens‐related ocular discomfort and dryness symptoms than nonusers. However, contact lens use was not independently associated with higher OSDI scores after adjustment for gender and daily screen exposure. These findings suggest that contact lens wear may be associated with lens‐specific ocular discomfort, but they do not establish an effect of contact lens use on the clinical severity of DED. Objective ocular‐surface assessments were not performed, and residual confounding by other unmeasured factors cannot be excluded. These findings highlight the importance of assessing DES and contact lens‐related discomfort among university students who wear contact lenses. Appropriate lens‐wearing practices and regular eye examinations may help identify and manage ocular discomfort in this population. Prospective studies are needed to clarify the temporal relationship between contact lens use and ocular symptoms and to determine whether these symptoms correspond to objectively measured ocular‐surface abnormalities. Future studies should combine validated symptom questionnaires with appropriate objective tear‐film and ocular‐surface assessments.

## Author Contributions

L.E.: Data collection, manuscript writing and editing. A.Y.: Data collection, manuscript writing and editing. F.D.: Data collection, manuscript writing and editing. B.Z.: Data collection, manuscript writing and editing. H.A.: Project concept, project supervision, data analysis validation, manuscript writing and editing. M.G.: Project concept, project supervision, data analyses validation, manuscript writing and editing manuscript submission. M.R.: Data analyses, data analyses validation, manuscript writing and editing. M.A.: Data analyses, data analyses validation, manuscript writing and editing. I.M.B.: Data analyses, data analyses validation, manuscript writing and editing.

## Funding

No funding was received for this manuscript.

## Conflicts of Interest

The authors declare no conflicts of interest.

## Data Availability

The data that support the findings of this study are available from the corresponding author upon reasonable request.
